# Exploratory deep-learning-driven early risk stratification of significant neurological injury in pediatric extracorporeal membrane oxygenation

**DOI:** 10.3389/fdgth.2026.1857835

**Published:** 2026-08-21

**Authors:** Haiyang Tang, Molly McGetrick, James Hwang, Salman Sohrabi, Lakshmi Raman, Hanli Liu

**Affiliations:** 1 Department of Bioengineering, University of Texas at Arlington, Arlington, TX, United States; 2 Department of Pediatrics, Division of Cardiology, Heart Center, Children's Health, UT Southwestern Medical Center, Dallas, TX, United States; 3 Department of Pediatrics, UT Southwestern Medical Center, Dallas, TX, United States

**Keywords:** deep learning, electroencephalography (EEG), extracorporeal membrane oxygenation (ECMO), feature fusion, significant neurological injury (SNI)

## Abstract

**Objective:**

To develop and examine a multimodal deep-learning framework utilizing clinical variables with and without time-dependent electroencephalogram (EEG) spectral features for predicting significant neurological injury (SNI) in pediatric patients with extracorporeal membrane oxygenation (ECMO).

**Methods:**

Data were collected from 73 pediatric ECMO patients. After excluding patients who had missing or discontinuous temporal data within 72 h after EEG initiation, 43 patients with usable EEG time-series data were analyzed. Neurological injury severity was quantified using a neuroimaging score (NIS) derived from post-cannulation imaging, with SNI defined as NIS ≥ 8. Model inputs included six clinical variables and six EEG features representing relative delta (1–4 Hz) and theta (4–8 Hz) power from the left, right, and bilateral hemispheres. Down-sampled EEG power signals were segmented into multiple 30-minute windows. A deep-learning fusion framework with modality-specific encoders was developed for SNI prediction, followed by SHapley Additive exPlanations (SHAP)-based feature attribution analysis.

**Results:**

At the patient level, the proposed multilayer perceptron model used only for clinical variables achieved the highest overall performance with an area under the curve (AUC) of 74.29%. The Wilcoxon signed-rank test was performed and showed no statistical significance in model performance between the clinical-only and fusion model. SHAP analysis indicated that clinical variables were the primary contributors, not the EEG inputs, to SNI prediction.

**Conclusion:**

A multilayer perceptron neural network used with six clinical inputs demonstrated moderate performance in identifying SNI among pediatric ECMO patients; integration with EEG-derived spectral features did not significantly enhance the performance. However, this conclusion is highly exploratory and dependent on selected EEG inputs, thus needing to be valid with a larger patient cohort, appropriate EEG feature selections, and assess cross-site generalizability.

## 1 Introduction

Pediatric extracorporeal membrane oxygenation (ECMO) utilization has risen sharply over two decades (1). Though ECMO boosts survival in critically ill children, in-support neurological injury drives substantial morbidity and mortality (2–6). Neurological complications affect 20%–30% of pediatric ECMO patients, and injured neonates face double the mortality risk (4, 7). Long-term follow-up reveals 10%–50% of survivors exhibit cognitive or neurodevelopmental deficits, with 40% developing at least one disability by school age (2, 3, 5, 6). These data highlight an urgent demand for early, objective risk stratification tools to enable prompt targeted interventions. Previous research largely evaluated isolated clinical predictors of brain injury (8, 9), yet discrete clinical variables alone lack sufficient predictive power for heterogeneous ECMO cohorts.

Prior studies have shown that pediatric cohorts demonstrate multiple EEG markers correlated with poor neurological prognoses: attenuated amplitude, low-voltage or inactive background, absent reactivity, and interhemispheric asymmetry. For hypoxic-ischemic encephalopathy neonates, low-voltage and isoelectric EEG tracings strongly predict impaired neurodevelopment (10), while sustained low-voltage interburst intervals also signal adverse outcomes (11, 12). Reduced EEG complexity predicts unfavorable results in abusive head trauma (13), and lost reactivity during coma indicates poor prognosis (14). Among pediatric ECMO patients, EEG abnormalities such as asymmetrical activity, suppression ratios, and interhemispheric power disparities correlate with neuroimaging lesions and acute brain injury (7, 15, 16). Despite the proven prognostic utility of EEG biomarkers, existing literature remains largely descriptive and analyzes EEG signals separate from clinical covariates. This restricts their integration into rigorous multivariable risk models and motivates multimodal frameworks combining EEG, physiological, imaging, and clinical data.

Machine learning (ML) is a branch of artificial intelligence that uses algorithms to discover patterns in clinical data. ML approaches can model complex, non-linear relationships between clinical and physiologic data, rendering it an attractive tool for neurological risk stratification in pediatric ECMO. Deep learning (DL), a subfield of ML, excels at extracting hierarchical features from raw inputs and classifying data based on those features. DL can also integrate multiple variables, model their interactions, and generate predictions for diverse feature combinations. Some studies have applied machine learning or DL to neurological risk stratification for ECMO patients with varying levels of accuracy (17, 18). Shah et al. built a hybrid recurrent-convolutional neural network using 35 physiological metrics (e.g., heart rate) from pediatric ECMO patients to predict significant neurological injury (SNI), achieving an area under the receiver operating characteristic curve (AUROC) of 0.76 (17). AUROC quantifies binary classifier performance by balancing true and false positive rates across cutoffs. Kalra et al. trained tree-based models on demographics, pre-ECMO laboratory values, hemodynamics, and 24-hour post-cannulation ECMO parameters, yielding an AUROC of 0.72 (18). Deng et al. used a random forest model with 15 curated clinical/ECMO variables and reached an internal AUROC of 0.912 and external AUROC of 0.807 (19). While these models attained moderate AUROC values, prior work solely utilized routine clinical covariates without incorporating continuous dynamic EEG time-series that capture real-time cerebral function (17, 18). Additionally, existing analyses evaluated clinical or physiological data separately instead of fusing both modalities in one unified predictive framework.

This exploratory study hypothesized that a multimodal deep learning (DL) framework integrating clinical variables and time-varying EEG spectral features would yield superior performance for identifying SNI in ECMO patients, compared with a model using clinical data alone. To test this hypothesis, we developed and evaluated three distinct DL models: a clinical-only model, a multimodal model combining clinical variables with temporal EEG spectral features extracted from continuous monitoring acquired within 72 h of EEG initiation, and an EEG-only model using solely the temporal spectral features. We further quantified the relative predictive weight of each clinical and EEG-derived feature to enhance model interpretability and guide refined model development for future clinical translation. In the Discussion section, we discussed core DL-based risk stratification results for pediatric ECMO-associated SNI according to whether the hypothesis was validated or rejected.

## 2 Materials and methods

### 2.1 Patient selection and SNI labeling

A total of 73 pediatric patients were included in this study. All patients were managed according to standard institutional sedation protocols at Children's Medical Center Dallas, which typically included narcotic infusion, dexmedetomidine, and benzodiazepine if additional sedation was needed. Time zero was defined as initiation of ECMO support. For each patient, a neuroimaging score (NIS) was obtained based on a post-ECMO cannulation brain (CT or MRI) image. This score was calculated using a previously validated methodology (9, 20). If a patient had multiple post-ECMO cannulation images, the image with the highest assigned NIS score was used for outcome assignment. Thus, the SNI label reflects the maximum radiographic injury severity identified during the post-cannulation imaging window, rather than necessarily indicating that the injury first occurred after EEG monitoring began. An NIS ≥ 8 was defined as SNI, and an NIS < 8 as no SNI (No-SNI) from a previous validated measure (9, 20). In our composite dataset, 20 patients (27.4%) had SNI (NIS ≥ 8) and 53 (72.6%) had No-SNI (NIS < 8).

### 2.2 Selection and definition of clinical variables

Multiple clinical variables were collected from each patient. For this proof-of-principle study, we selected six variables that were consistently available across the cohort and clinically relevant to neurologic risk during ECMO, namely age, sex, diagnosis, ECMO type, weight, and body surface area (BSA). These variables were defined as follows:

1) Age: Age was recorded as the number of days from birth to ECMO cannulation. In this cohort, age ranged from 0 to 6,973 days, corresponding to approximately 19 years.
2) Sex: Sex was recorded as either male or female and encoded by integer 1 or 2, respectively.
3) ECMO type: ECMO type indicates the cannulation configuration used for cardiopulmonary support. VV denotes veno-venous ECMO, whereas VA denotes veno-arterial ECMO.
4) Diagnosis: Diagnosis reflects the primary clinical indication documented prior to ECMO cannulation. Diagnoses were group into five categories: (i) cardiac arrest, (ii) cardiogenic shock, (iii) sepsis, (iv) pulmonary hypertension, and (v) respiratory failure.
5) BSA: BSA is the estimated surface area of the human body. It was computed based on the weight in kilograms (kg) and height (cm) of the patient using the formula: BSA=
6) Weight: The weight of the patient in kilograms at the time of ECMO cannulation.
  $$"\mathrm{BSA}"=\sqrt{\left((\mathrm{Weight}\;\text{·}\mathrm{Height}\right)/3600)}$$

While many clinical factors may influence outcomes, these six variables were selected because they were consistently available and captured key demographic, anthropometric, clinical, and ECMO-related characteristics that may influence neurologic risk. For example, ECMO type reflects differences in cannulation strategy and underlying physiology, while primary diagnosis captures the clinical context leading to ECMO support. Age, weight, and BSA account for developmental and body-size differences that may affect both clinical course and EEG acquisition. Additional patient characteristics are summarized in Supplementary Table S1, Section A of Supplementary Material.

### 2.3 EEG recording and data processing

Twenty one scalp electrodes were placed using the international 10–20 system (21). The timing of the EEG recording initiation is consistent with published neuromonitoring guidance for neonatal and pediatric ECMO (22). Specifically, per institutional protocol, EEG monitoring was initiated around 6–12 h after ECMO cannulation when clinically feasible and lasted for 1–7 days. To enable an early prediction of SNI, we took only the first 3 days of EEG recordings after EEG initialization as the data for model training, validation, and testing. In this step, 12 patients were excluded because they had missing data within the 3 days for an un-noted reason after EEG initiation. Detailed information on the timing of ECMO cannulation, the time from ECMO cannulation to EEG initiation, and time from ECMO cannulation to first neuroimaging are provided in Supplementary Table S1, Section A of Supplementary Material.

Quantitative EEG data were analyzed using the Persyst Analysis Software, Version 13 (Persyst Development Corporation, San Diego, CA) (23). In general, Persyst processes continuous EEG time-series data by segmenting the signals into short, overlapping windows and applying frequency-domain analysis using the Fast Fourier Transformation to estimate time-resolved power spectra. Spectral power is then integrated within canonical EEG frequency bands (e.g., delta, theta, alpha, and beta) and temporally compressed through averaging and down-sampling to facilitate visualization of long-term trends over hours. Relative spectral power is calculated by normalizing band-specific power to the total spectral power, thereby emphasizing changes in spectral composition while reducing sensitivity to absolute amplitude variations. For this study, Persyst was configured to exclude periods of photic stimulation, electrographic seizures and epochs when the calculated total power was equal to 0. The EEG data were resampled at a frequency of 64 Hz using 2-second windows. After processing, 8 overlapping segments were averaged to generate total and relative power outputs for each frequency band every 8 s. The Persyst-processed data used in this study consisted of six time-resolved relative EEG power features, as listed below:

1) Time-dependent relative delta power, defined as the delta-band power between 1 and 4 Hz normalized to total power between 1 and 20 Hz, calculated for the left hemisphere, right hemisphere, and bilateral hemispheres.
2) Time-dependent relative theta power, defined as the theta-band power between 4 and 8 Hz normalized to total power between 1 and 20 Hz, calculated for the left hemisphere, right hemisphere, and bilateral hemispheres.

Data were reported by Persyst at frequencies of 0.125 Hz for delta power and 0.1 Hz for theta power.

A 64-Hz resampling rate preserves clinically relevant lower-frequency EEG activity, including delta and theta frequencies, while attenuating higher-frequency noise and artifacts above 32 Hz. This approach improves computational efficiency, enables faster real-time quantitative EEG (qEEG) trend generation for ICU monitoring, and simplifies signal processing by aligning with power-of-2 data segmentation commonly used in qEEG algorithms.

**Figure 1:**
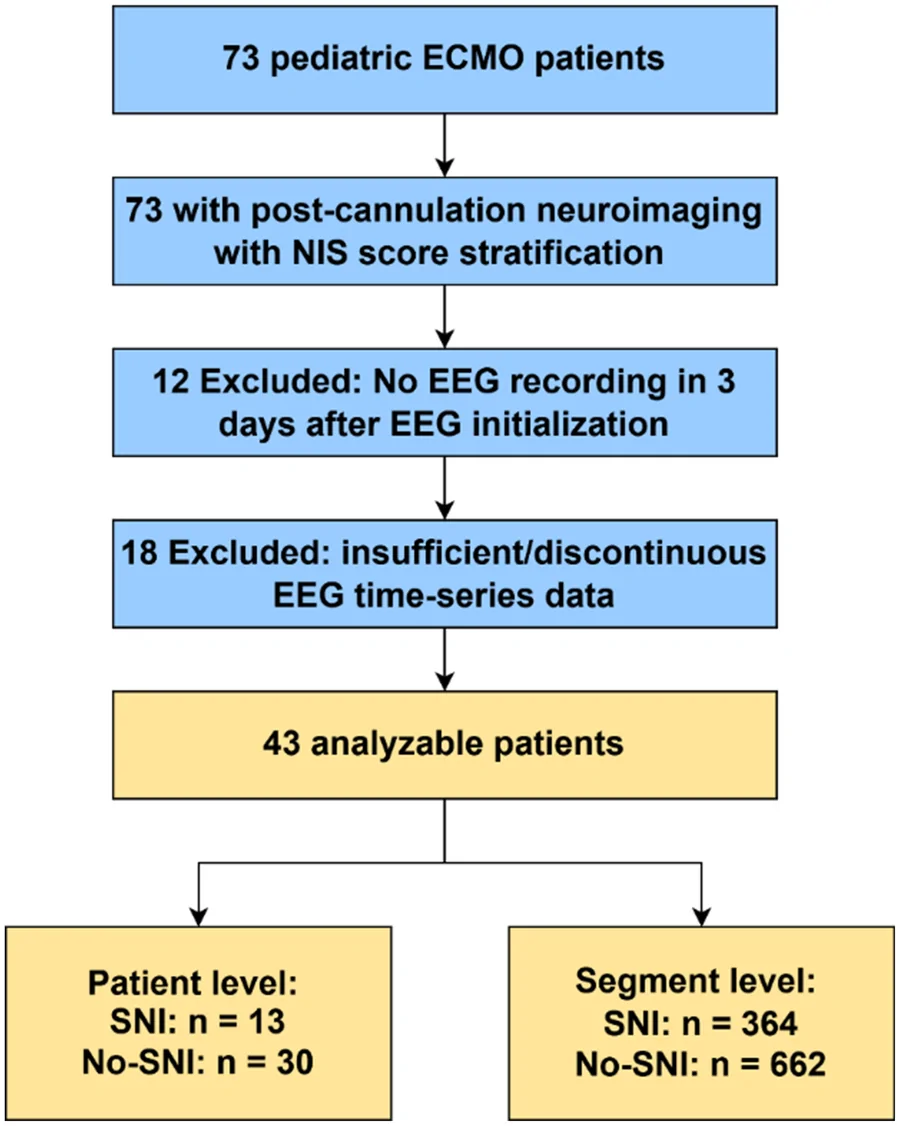
Flowchart of data preprocessing and segmentation. A total of 73 patients were included in the study, and their post-cannulation neuroimaging scores were available. 12 patients were excluded because they did not have EEG recordings within 3 days (≤ 72 h) of the EEG initiation. Then, 18 patients were excluded after the respective time series data were segmented into 30-min non-overlapping time periods. Thus, 43 patients were included in the machine learning analysis. The blue boxes show the preprocessing steps, and the yellow boxes show the final data grouping and segmentation.

**Figure 2:**
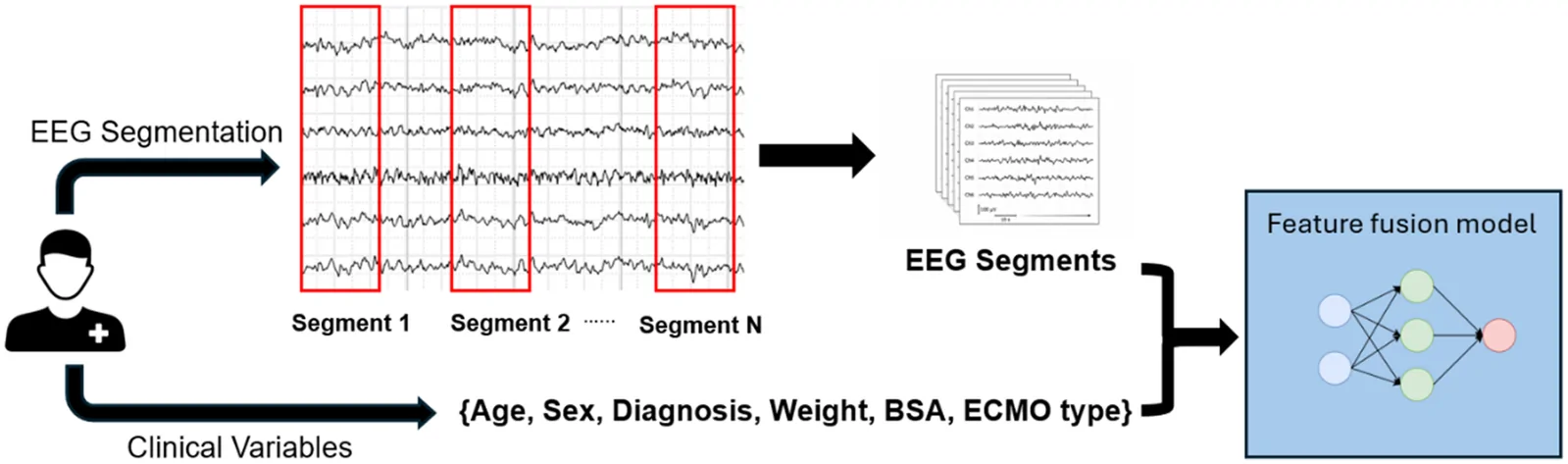
The pipeline in this work. A patient's EEG recording will be segmented into segments of duration exactly 1,800 seconds, then fed into deep learning models along with clinical variables.

Next, the Persyst-derived time-dependent relative delta and theta power series were then segmented into multiple continuous 30-minute (1,800 second) segments to increase the number of samples available for building the deep learning model. Each segment was required to satisfy the following two conditions: (1) a duration of exactly 30 min and (2) no missing values within the segment (i.e., no data labeled with NaN or 0). Because some EEG recordings contained intermittent gaps, continuous 30-min segments could not be extracted for all patients. This processing step filtered out low-quality temporal data and further excluded 18 patients, resulting in a final analytic cohort of 43 patients, including 13 with SNI and 30 without-SNI (no-SNI), as shown in Figure 1. These EEG segments, along with clinical variables, served as inputs to the machine learning models, as illustrated in Figure 2.

By design, all SNI and No-SNI segments were derived from patients classified as SNI and No-SNI, respectively. In total, 1,026 segments were extracted, including 364 SNI segments and 662 No-SNI segments. Detailed descriptions of the clinical variables and EEG recordings included in this study are provided in Supplementary Tables S2 and S3 (Section A of Supplementary Material).

For each patient, qualitative EEG was reviewed by a trained epileptologist to assess for electrographic seizure activity. The overall incidence of seizures was low. Among the 1,026 segments from 43 patients, only one segment contains 15 s of electrographic seizure activity, indicating that the potential impact of seizure activity on the study results was considered negligible.

### 2.4 Institutional review board approval

Data was collected in accordance with the ethical standards of the Committee for the Protection of Human Subjects at the University of Texas Southwestern Medical Center and Children's Health in Dallas, Texas. This study was approved by the UT Southwestern Institutional Review Board (protocol STU-2021–0095, PELICAN). Written informed consent (and assent when applicable) was obtained from participants and/or their legally authorized representatives.

### 2.5 Proposed fusion framework

**Figure 3:**
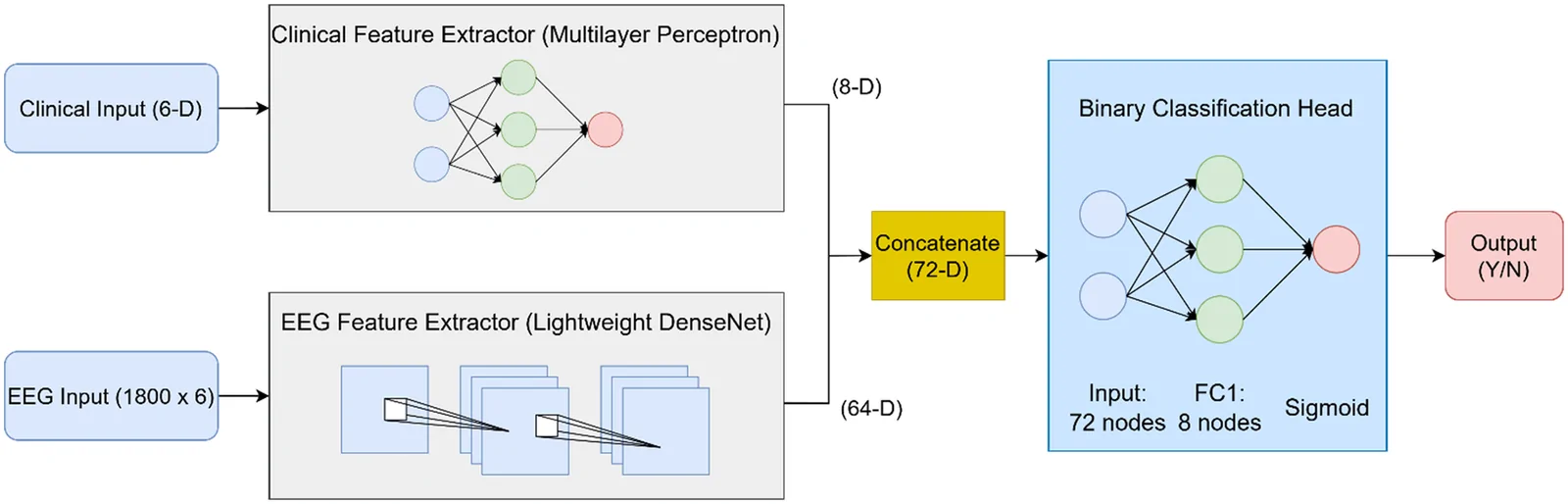
Overview of the proposed fusion framework for SNI classification. The model consists of two extractors that process EEG and clinical inputs, respectively. The EEG input is a 1,800   ×   6 matrix, and the clinical input is a 6-D vector. Each input is passed through its own feature extractor to produce a corresponding feature vector. These two vectors are then concatenated along the feature dimensions and fed into a binary classification head that outputs the final SNI classification. FC: fully connected layer.

The proposed fusion framework combines the selected continuous 30-minute (1,800-second) relative power segments with clinical variables to classify SNI versus No-SNI. As shown in Figure 3, the fusion framework has three components: (i) an EEG feature extractor, implemented as a DenseNet-style (24) Convolutional neural network (CNN) with bottlenecks to learn temporal–spectral patterns; (ii) a clinical feature extractor, implemented as a multilayer perceptron (MLP) to model clinical covariates; and (iii) a binary classification head. The latent vectors from the EEG and clinical branches are concatenated and passed to an MLP head that outputs the final prediction. Detailed hyperparameter settings for each component are provided in Table S6 (Section B of Supplementary Material).

**Figure 4:**
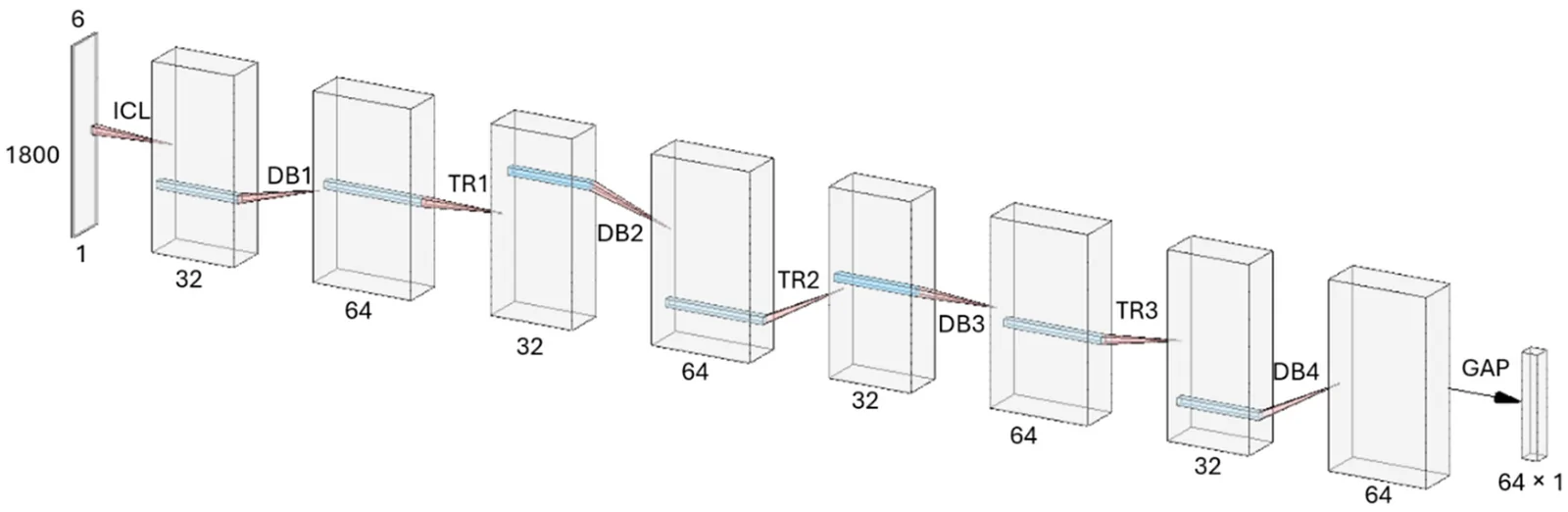
Structure of the proposed lightweight denseNet-style EEG feature extractor. GAP: global average pooling layer. DB, DenseBlock; TR, transitional layer. More detailed information is available in Supplementary Table S6.

(i) EEG feature extractor. The EEG feature extractor is a DenseNet-style CNN with four dense blocks, as shown in Figure 4. The EEG input is a 1,800   ×   6 matrix: relative time-dependent delta (1–4 Hz/1–20 Hz) and theta (4–8 Hz/1–20 Hz) powers in the left, right, and bilateral hemispheres. The network begins with a 2D convolution (32 filters, 3   ×   3), followed by four DenseBlocks, each containing two Dense layers, where each Dense layer consists of one 1   ×   1 convolutional layer and one 3   ×   3 convolutional layer with ReLU activation. The growth rate (gr) is set to 16 within each DenseBlock. Transition layers between consecutive DenseBlocks downsample the feature maps by a ratio of 0.5. After the last DenseBlock, a global average pooling layer then converts the matrix into a 64-dimensional vector (64   ×   1). The 1   ×   1 convolutional layers in each dense block are called bottleneck (bn) layers. Despite using a DenseNet-style CNN, we also implemented several EEG feature-extraction architectures to compare performance across models.
(ii) Clinical feature extractor. Six variables were used as clinical inputs: age in days, diagnosis, ECMO type, BSA, weight, and sex. Each subject is represented by a 6-element vector. This branch consists of three fully connected layers with 8, 16, and 8 units each, producing an 8-dimensional output embedding, with ReLU activations applied after each layer to introduce nonlinearity.
(iii) Binary classification head. The 64-D EEG embedding and the 8-D Clinical embeddings are concatenated into a 72-D feature vector. We apply dropout (*p* = 0.2) immediately after concatenation, followed by two fully connected layers with 8 and 1 units, respectively. A final 1-unit dense layer with a sigmoid activation produces the binary probability.

### 2.6 Optimization strategy

We trained the model with binary cross-entropy as the loss function and saved the best model based on the lowest validation loss. We did not select the model based on the lowest validation accuracy because the dataset was small and imbalanced. High accuracy can be misleading, favoring the majority class and not guaranteeing the best final performance. Implementation and examination of the models

## 3 Implementation and examination of the models

### 3.1 Input preprocessing

The model ingests two types of input features: clinical variables and EEG segments. Due to the small dataset, we used ordinal encoding rather than one-hot encoding to transform categorical clinical variables into numerical values. To be more specific, for a feature with *n* categories, one-hot encoding expands it into an *n* dimensional binary vector, increasing the input dimension by *n* times. In contrast, ordinal encoding assigns a single integer label to each category, preserving the original feature dimension and making it more suitable for small datasets. In the preprocessing of clinical variables, age in days was binned into four ranges (0–30, 31–365, 366–1,825, >1,825) based on typical neurodevelopmental age ranges and was ordinal encoded as 0, 1, 2, and 3, respectively. Diagnosis was encoded into five categories mapped to integers 0–4 (cardiac arrest = 0, cardiogenic shock = 1, pulmonary hypertension = 2, respiratory failure = 3, and sepsis = 4). ECMO type was encoded as 0 or 1 (VA = 0, VV = 1). Sex was kept as recorded (male = 1, female = 2). The weight of each patient was rescaled by dividing by 100 to place it on a smaller numerical range. BSA was using the original values. No preprocessing was conducted on the EEG inputs because they were already normalized to relative power.

### 3.2 Cross-validation

Due to the relatively small sample size of 43 patients, we used 7-fold, patient-level, stratified cross-validation to ensure that each fold's training set included sufficient SNI patients. For each fold, we first partitioned patient IDs into training, validation, and test sets, then matched segments based on those IDs. Thus, during training, the model only saw segments from training-set IDs and never accessed segments from validation or test IDs.

Under the 7-fold cross-validation scheme, five of the seven test folds each contained 2 SNI patients and 5 No-SNI patients, one test fold contained 2 SNI patients and 4 No-SNI patients, and the remaining one test fold contained 1 SNI patient and 5 No-SNI patients. After splitting patients for the test set, 10% of the remaining patients will be used as the validation set. As a result, the validation set consistently included one SNI and three No-SNI patients. The patient distributions for all seven folds are summarized in Supplementary Table S4 (Section A of Supplementary Material).

Furthermore, to enable a fair comparison across models, we used three different random seeds (No. 1, No. 2, and No. 3) for patient splitting, and each was evaluated using 7-fold cross-validation. Across random seeds, the assignment of patient IDs to the training, validation, and testing sets varied due to stochastic partitioning. However, the number of patients in the training, validation, and testing sets remained fixed, ensuring that each seed yields the same number of patients in each split. In total, each model evaluation includes 21 training–validation–testing evaluations (7 folds   ×   3 seeds). The final performance of each model was obtained by aggregating the test results across the 21 evaluations. Specifically, for a given evaluation metric *i* (e.g., accuracy), the final aggregated value $i_{f}$ was computed as:

(1) $$i_{f}=\frac{1}{21}\sum_{r=1}^{21}i_{r}$$

where $i_{r}$ denotes the evaluation metric obtained in the r-th run, with r=1,…,21. Similarly, if we define the $\bar{i}$ to be the average value of *i* across 21 evaluations, the overall standard deviation (std) of *i* across 21 evaluations is then computed as:

(2) $$std=\sqrt{\frac{1}{21-1}\overset{21}{\underset{r=1}{\sum }}{\left(i_{r}-\bar{i}\right)}^{2}}$$

### 3.3 Ablation study

To quantify the relative contributions of each modality, we performed ablation experiments to examine whether EEG features or clinical variables alone could achieve comparable predictive accuracy. Accordingly, we defined three input settings:

1) EEG-only input: In this configuration, the model consisted solely of the EEG feature extractor and received only EEG segments as input. No clinical variables were provided to the model.
2) Clinical-data only input: In this configuration, the model included only a clinical feature extractor and received clinical variables as the sole input. No EEG data were provided to the model.
3) EEG + Clinical input (dual input): In this configuration, the model included both an EEG feature extractor and a clinical feature extractor. The EEG feature extractor varied across the evaluated architectures.

### 3.4 Models for comparison and implementation details

To better evaluate the performance of our fusion model, we ran several existing models using each of the three input settings. For the clinical-only input setting, we evaluated four other machine learning algorithms: Light Gradient Boosting Machine (LGBM) (25), K-Nearest Neighbors (KNN) (26), XGBoost (27), and gradient boosting (GB) (28). For the EEG-only input setting, we selected three other models that were designed for EEG processing: EEGNet (56), ShallowConvNet (29), and EEG-ITNet (30). For the dual-input setting (EEG + clinical), we paired each EEG-only feature extractor with the proposed MLP clinical feature extractor to enable a controlled comparison across the EEG feature extractor architectures. All deep learning models used for EEG feature extraction were implemented according to the standard implementation hyperparameters based on their publication.

To ensure comparability across models and input settings, all modeling experiments were conducted using 7-fold cross-validation with the same three random seeds. Consequently, each model was evaluated over 21 training–validation–testing evaluations (7 folds   ×   3 seeds). Final performance metrics were then obtained by aggregating results across all 21 evaluations according to Equations 1, 2.

All deep learning models were trained for 100 epochs in the 7-fold cross-validation using the same three random seeds. A learning rate scheduler with a patience of 10 epochs and a decay rate of 0.5 was applied to the deep learning model's training. To determine the best initial learning rate (ILR) and batch size, all the models were tested on three levels of learning rate, namely $10^{-3}$, $10^{-4}$, and $10^{-5}$ using a batch size of 4. After the best learning rate has been determined, the models will be tested at three other batch sizes: 32, 64, and 128. Finally, the best results out of the best learning rate and batch size will be reported. For the detailed information on the selected learning rate, batch size, and other hyperparameters, please refer to Supplementary Tables S7, S12 (Section B of Supplementary Material).

The Adam optimizer (31) with the standard momentum parameters were used. After model training, the model with the lowest validation loss was loaded for testing. All model training and testing were performed on Google Colab using A100 GPUs.

### 3.5 Evaluation metrics

The evaluation of model performance was conducted and reported at the patient level. Specifically, for each patient, all the predicted probabilities were averaged across the available segments to obtain a single patient-level prediction. These segment-averaged probabilities per patient were then pooled at the group level for the model performance.

AUROC was used as the primary evaluation metric in our experiments because it summarizes classification performance across all possible decision thresholds from 0 to 1, providing a threshold-independent assessment of discriminative ability. This offers a more comprehensive evaluation than the metrics computed at a single fixed threshold, such as 0.5.

In addition to AUROC, we computed and reported accuracy, precision or positive prediction value (PPV), recall or sensitivity), specificity, and F1-score (the harmonic mean of precision and recall):

$$\mathrm{Accuracy}=\frac{\mathrm{TP}+\mathrm{TN}}{\mathrm{TP}+\mathrm{TN}+\mathrm{FP}+\mathrm{FN}}$$

$$\mathrm{Precision}=\mathrm{PPV}=\frac{\mathrm{TP}}{\mathrm{TP}+\mathrm{FP}}$$

(3) $$\mathrm{Recall}=\mathrm{Sensitivity}=\;\frac{\mathrm{TP}}{\mathrm{TP}+\mathrm{FN}}$$

$$\mathrm{Specificity}=\frac{\mathrm{TN}}{\mathrm{TN}+\mathrm{FP}}$$

$$F1=2\cdot \frac{\mathrm{Precision}\cdot \mathrm{Recall}}{\mathrm{Precision}+\mathrm{Recall}}$$

where TP = true positive, TN = true negative, FP = false positive, FN = false negative. Note that the F1 score, defined as the harmonic mean of precision (positive predictive value) and recall (sensitivity), was used to evaluate the balance between false-positive and false-negative classifications. F1 is an average that strongly penalizes imbalance between the two quantities, ensuring that both precision and recall must be high simultaneously.

To ensure reliable performance evaluation and prevent data leakage, Youden's J-optimal threshold was determined exclusively from the training data within each cross-validation fold and then applied to the corresponding held-out test set. Specifically, after model training in each fold, the optimal threshold was selected using the training set and fixed before evaluating threshold-dependent metrics, including accuracy, precision, recall, and specificity, on the test set. The resulting performance metrics were averaged across 21 runs, corresponding to 7-fold cross-validation repeated with three random seeds, according to Equations 1, 2, to obtain the final model performance. Note that AUROC is a threshold-independent metric and therefore remains unchanged regardless of the threshold-selection strategy.

### 3.6 Analysis of feature importance using SHAP

SHapley Additive exPlanations (SHAP) is a method used to identify how strongly each clinical or physiological factor influences a deep learning model's prediction, thereby helping clinicians understand the basis of the model's decision. Specifically, the SHAP framework was implemented to quantify the importance of the input features, using the DeepSHAP subpackage (shap. DeepExplainer) (32). This approach was selected because several clinical variables were coded as integers, where a value of 0 represented a valid clinical category rather than a missing or null state. Attribution approaches that assume an all-zero baseline would therefore treat this valid category as the reference condition, potentially leading to biased or misleading interpretations. In contrast, DeepSHAP leverages a background distribution constructed from representative samples in the training and validation sets and computes the SHAP values relative to these representative examples. This property makes DeepSHAP well-suited for our mixed-input architecture consisting of EEG time-series data and tabular clinical variables, without requiring an artificial ‘blank' baseline category.

In general, for feature *i*, the SHAP value for input *x* can be written:

(4) $$\phi_{i}(x)=\underset{S\subseteq \{1,\ldots ,M\}\setminus \{i\}}{\sum }\frac{|S|!(M-|S|-1)!}{M!}(v(S\cup \{i\})-v(S)),$$

where *M* is the number of features, *S* is a subset of features excluding *i*, and |S| is its size. v(S) denotes the model output when only the features in *S* are present, and the remaining features are integrated with respect to a background distribution (32).

In terms of the DeepSHAP approach, it approximates SHAP values by comparing the model output at *x* to the model output at background samples *z* and using DeepLIFT multipliers, a finite-difference, reference-based backprop rule to attribute the difference:

(5) $$\phi_{i}(x)\approx \frac{1}{K}\overset{K}{\underset{k=1}{\sum }}(x_{i}-z_{i}^{\left(k\right)})\;m_{i}(x,z^{\left(k\right)}).$$

Intuitively, for a categorical feature, DeepSHAP averages the feature's contribution computed relative to each background sample $z^{\left(k\right)}$ to the sample's value. Averaging across *K* background samples provides an empirical approximation of the expected contribution of each feature relative to the representative patient examples. Background expectations in the SHAP value function v(S). DeepLIFT multiplier, represented as $m_{i}(x,z^{\left(k\right)})$, provides the magnitude of attribution for feature changes. Note that the DeepSHAP background is a subset of real samples drawn from the training and validation sets (32).

In our experiments, the importance of the six clinical variables was computed using equations. (5), which was implemented using a Python package (shap. DeepExplainer). To quantify the overall influence of the clinical input variables, we used the sum of the absolute values to represent the magnitude of each variable's impact on the risk stratification model. For the EEG input, we assessed the channel-wise importance of the six EEG band power metrics. Let $S_{n,t,c}\;$ denotes the DeepSHAP value for patients n∈{1,…,N}, time t∈{1,…,T}, and channel c∈{1,…,C}. Point-wise importance is $abs(S_{n,t,c})$, obtained from Equation 5. We then summed the absolute DeepSHAP values over all time points to represent the importance of an EEG band power metric for one patient. The final importance score was computed by averaging the importance of each channel across all the patients from 1 to N to obtain the importance score $T_{c}$ for an input EEG band power, as shown in Equation 6.


(6) $$T_{c}=\frac{1}{N}\overset{N}{\underset{n=1}{\sum }}\overset{T}{\underset{t=1}{\sum }}|S_{n,t,c}|.$$

SHAP values were computed for six EEG band-power features: relative delta and theta power each normalized to total 1–20 Hz power extracted from the left, right, and bilateral hemispherical regions. To balance computational efficiency and estimation stability, we set the number of background samples to 64 when computing SHAP values for all features. final SHAP values for both EEG features and clinical variables were obtained by aggregating results across all 21 training–validation–testing evaluations using Equations 1, 2.

### 3.7 Statistical testing to compare model performance

To test our hypothesis, it was necessary to statistically compare the model performance between the fusion and clinical-only models. Specifically, a paired Wilcoxon signed-rank test was used to compare the proposed clinical-only MLP model with the dual-input fusion model using paired patient-level AUROC values over 21 runs.

## 4 Results

### 4.1 Model performance across three different input settings

Table 1 summarizes the patient-level model performance across three input settings: EEG only, clinical data only, and combined both, for different models. As shown in the Table, in the clinical-data-only setting, the proposed MLP method achieved an AUROC of 74.29% and an accuracy of 72.45%, outperforming all competing models and all input modalities. Among the models, KNN achieved the highest recall, accuracy (same as the proposed MLP model), and F1 score across all input modalities, while LGBM achieved the highest specificity across all input modalities. Because the proposed MLP achieved the highest AUROC in the clinical-data-only experiments, it was selected as the clinical feature extractor for all dual-input (EEG + clinical) models.

**Table 1:** Patient-level model performance is reported as mean ± standard deviation in seven-fold cross validations across 43 patients, with three different random seeds. ‘Proposed' denotes the fusion model introduced in this study. For each metric. Accuracy, precision (i.e., positive prediction value, PPV), recall (i.e., sensitivity), and specificity are calculated at Youden's J–optimal threshold using the training set for each respected fold.

Input Modality	Model	AUROC (%)	Accuracy (%)	Precision or PPV (%)	Recall or Sensitivity (%)	Specificity (%)	F1 (%)
Clinical only	KNN	72.14 ± 17.35	72.45 ± 15.19	60.00 ± 29.99	61.90 ± 34.17	78.33 ± 19.96	54.31 ± 28.66
	LGBM	66.13 ± 29.98	69.50 ± 18.07	52.38 ± 40.27	33.33 ± 38.83	86.67 ± 19.48	32.54 ± 37.26
	XGBoost	63.87 ± 26.48	59.86 ± 15.49	34.74 ± 21.17	52.38 ± 39.27	64.76 ± 20.03	38.05 ± 26.87
	GB	63.21 ± 23.62	67.23 ± 20.41	51.30 ± 39.72	45.24 ± 40.55	76.90 ± 28.80	40.98 ± 35.00
	Proposed	74.29 ± 25.66	72.45 ± 14.30	56.85 ± 31.75	54.76 ± 37.50	80.71 ± 17.81	48.28 ± 31.32
EEG only	ShallowConvNet	68.81 ± 23.85	55.10 ± 19.76	39.13 ± 27.45	59.52 ± 42.59	54.52 ± 38.11	37.17 ± 26.64
	EEGNet	63.33 ± 22.85	58.62 ± 16.41	36.75 ± 22.66	50.00 ± 34.50	61.90 ± 28.05	37.80 ± 23.57
	EEG-ITNet	59.29 ± 27.43	56.80 ± 22.12	43.78 ± 34.46	50.00 ± 37.80	59.05 ± 35.78	38.44 ± 26.88
	Proposed	51.90 ± 27.53	52.61 ± 17.63	27.78 ± 24.26	42.86 ± 44.42	57.38 ± 36.92	26.01 ± 28.07
EEG + Clinical (EEG models integrated with a clinical-data MLP)	ShallowConvNet	63.81 ± 21.14	67.23 ± 20.07	58.81 ± 36.31	42.86 ± 41.65	79.05 ± 29.14	36.62 ± 34.53
	EEGNet	73.45 ± 19.86	69.39 ± 19.96	55.37 ± 34.55	54.76 ± 37.50	75.95 ± 25.76	48.14 ± 33.89
	EEG-ITNet	69.40 ± 28.19	69.16 ± 15.32	54.33 ± 32.66	45.24 ± 40.55	80.48 ± 22.78	38.59 ± 32.92
	Proposed	70.83 ± 24.70	72.22 ± 13.91	59.38 ± 32.26	42.86 ± 35.48	84.29 ± 16.85	41.43 ± 31.72

In the EEG-only setting, the standard ShallowConvNet achieved an AUROC of 68.81%, placing it among the best-performing EEG-only deep learning models. Overall, the differences in AUROC among the EEG-only models were modest, suggesting that EEG-derived features alone provided limited discrimination in this cohort.

In the dual-input setting, the proposed MLP was used as a unified clinical feature extractor for all comparative models. Under this configuration, the EEGNet achieved an AUROC of 73.45%, and the proposed fusion model (MLP + DenseNet-style CNN; see Figure 3) achieved an AUROC of 70.83%. The AUROCs of both EEGNet and the proposed fusion model in the dual-input setting are lower than the AUROC of the proposed MLP model in the clinical-only setting.

Moreover, as shown in Table 1, the mean differences between the proposed clinical-only model and the proposed EEG + clinical fusion model appear relatively small for several metrics, with relatively large standard deviations. To examine the statistical significance between the two models, a paired Wilcoxon signed-rank test was performed between the two models using paired patient-level AUROC values across 21 runs. The results showed that the mean *Δ*AUROC (clinical-only model minus fusion model) was +0.035, favoring the clinical-only model, but not statistically significant (*p* = 0.79).

These statistical results contradict our primary hypothesis: our deep learning fusion model combining clinical covariates and time-varying EEG spectral features showed no significant performance improvement over the clinical-only model for identifying SNI in pediatric ECMO patients. Nevertheless, this preliminary observation is highly conditional given the exploratory nature of this work; nuanced, multifactorial interpretations are provided in the subsequent Discussion section.

### 4.2 Feature importance or impact analysis

Although our primary hypothesis was not proved, quantifying each feature's independent predictive weight, particularly EEG-derived signals, remains clinically meaningful and scientifically insightful. We therefore applied SHAP analysis to quantify the predictive influence exerted by six clinical covariates and six EEG band-power time-series features.

**Figure 5:**
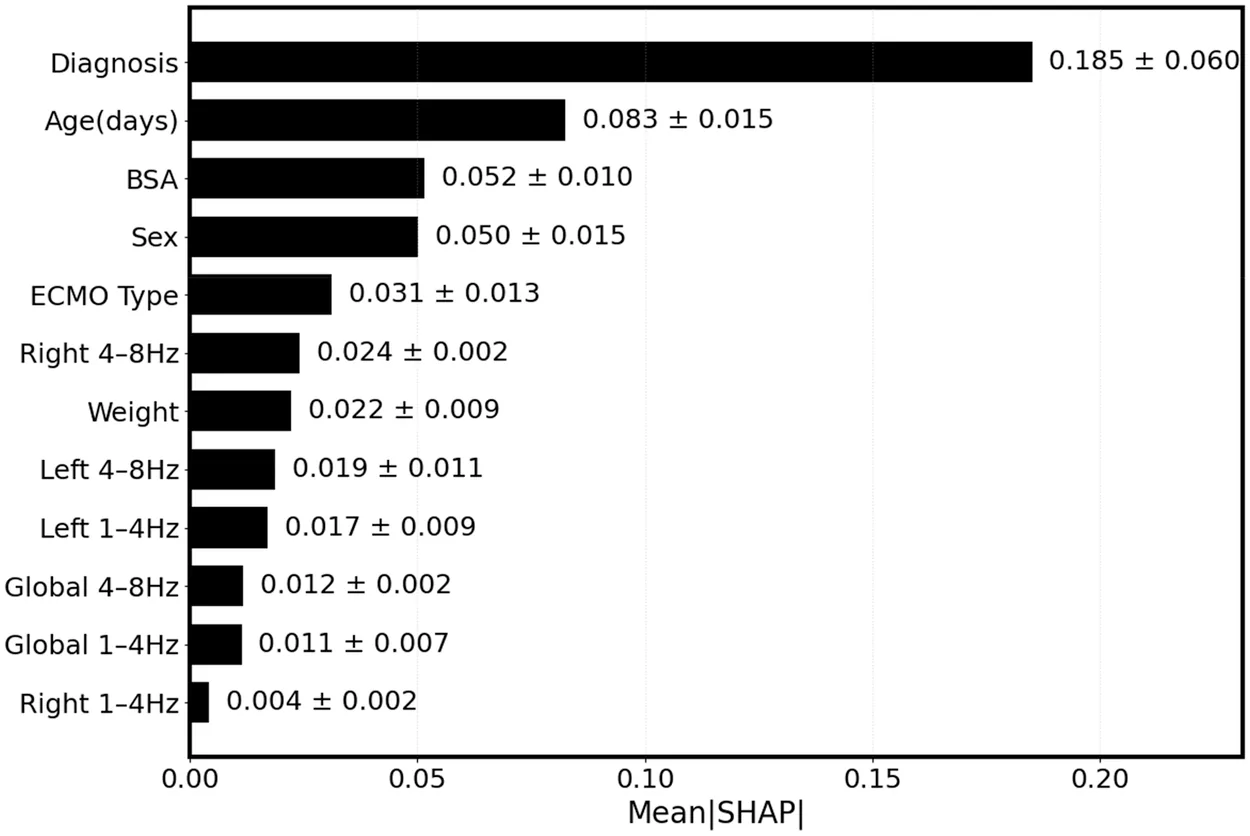
Mean absolute SHAP values of the input 6 clinical variables and EEG recordings. The feature of importance is plotted in descending order.

**Figure 6:**
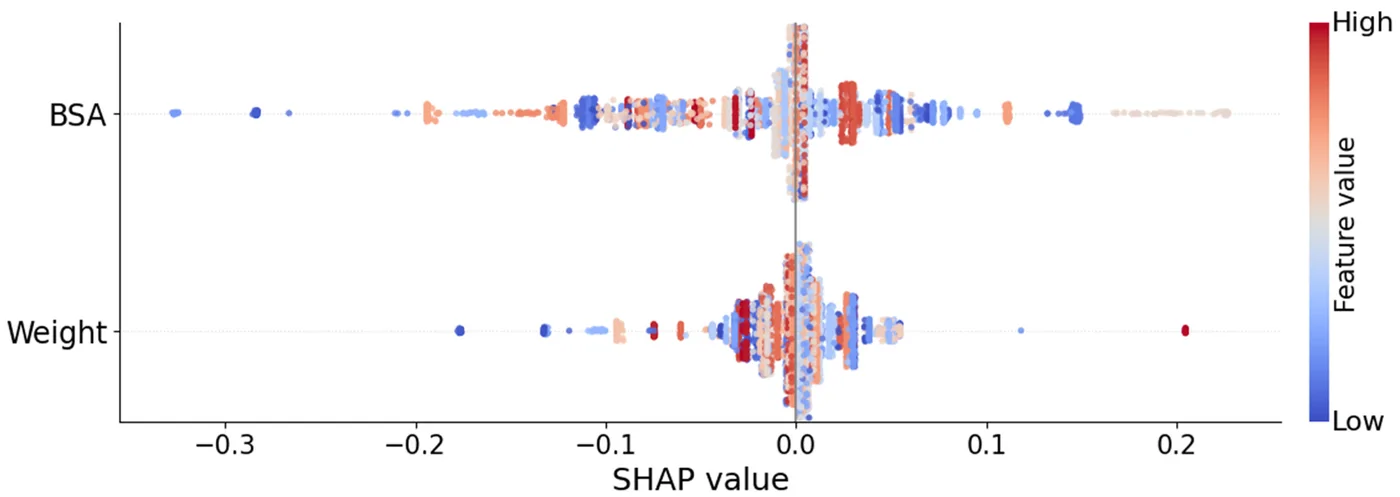
The effect of BSA and weight on model predictions. Each point represents one segment result.

Figure 5 summarizes the importance of features for six clinical variables. It shows that diagnosis has the highest mean absolute SHAP score among all clinical predictors, followed by age, BSA, sex, and ECMO type. Table 2 lists the respective sub-feature impacts within each clinical feature group. Among the diagnostic categories, cardiac arrest and cardiogenic shock were the strongest contributors toward higher model-estimated SNI risk, with SHAP values of +0.409 and +0.387, respectively, whereas other diagnostic categories (excluding cardiogenic shock) generally shifted the model output towards lower estimated SNI risk. Sex contributed minimally to model output, with both male and female sex associated with slightly positive SHAP values. Regarding age, age in 366–1,825 days (1 year - 5 years) shows the strongest positive contribution to SNI risk among different age ranges (SHAP = + 0.014), whereas age in 31–365 days (1 month to 1 year) shows the strongest contribution to lower estimated SNI risk among different age ranges (SHAP = −0.090). For ECMO type, VA has a positive contribution to SNI risk (SHAP = + 0.016), while VV shows a negative contribution to SNI risk (SHAP = −0.100). Figure 6 shows the effects of BSA and weight on model output, indicating that no obvious trend is evident regarding the values of BSA and weight and their impact on estimated SNI risk.

**Table 2:** SHAP analysis based on the proposed framework. Mean ± std of seven folds. The highest value in each category is bold and underlined.

Clinical Features	Category	Impact on estimated SNI risk
Diagnosis	Cardiac Arrest	+0.409 ± 0.129
	Cardiogenic Shock	+0.387 ± 0.309
	Pulmonary Hypertension	−0.086 ± 0.006
	Respiratory Failure	−0.101 ± 0.045
	Sepsis	−0.120 ± 0.040
Sex	Male	+0.018 ± 0.014
	Female	+0.001 ± 0.006
Age (days)	0–30 (1 month)	−0.068 ± 0.115
	31–365 (1 month to 1 year)	−0.090 ± 0.105
	366–1,825 (1 year - 5 years)	+0.014 ± 0.005
	>1,825 (> 5 years)	+0.010 ± 0.017
Type	VA	+0.016 ± 0.005
	VV	−0.100 ± 0.061

Category-level effects were not presented for the EEG predictors because the EEG inputs were continuous time-series features derived from relative delta and theta power rather than discrete, categorical variables. Therefore, their contributions are not naturally interpretable in terms of individual categories, similar to diagnostic and demographic variables. Instead, EEG feature importance was summarized across time and hemispheric regions using aggregated SHAP values.

## 5 Discussion

In this single-center study of neonatal and pediatric ECMO patients, we developed and evaluated a multimodal deep neural network that integrates clinical variables with EEG-derived spectral features for patient-level SNI risk stratification. The proposed MLP model using clinical-only input modality achieved an AUROC of 74.29% across seven-fold cross-validation testing, representing the highest discriminative performance among the evaluated deep-learning baselines. After a statistical analysis, we also found that the fusion of EEG with clinical data did not significantly improve the model performance. Although our results did not prove our primary hypothesis, we can still gain several key scientific insights from this exploratory study, which can be helpful to guide future DL model designs, more appropriate patient selections and EEG spectral selections, and multifactorial interpretations as discussed below.

### 5.1 Clinical significance of predicting SNI for pediatric ECMO patients

In routine ECMO care, neuroimaging via CT/MRI is generally performed weeks after decannulation to screen brain injury from primary disease or ECMO complications. While these scans support diagnosis and risk prediction, portable bedside noninvasive tools for early SNI detection are urgently needed. Early identification of at-risk patients enables prompt clinical intervention and optimized neurological care for this vulnerable cohort.

Continuous EEG provides a practical bedside method for assessing cerebral function during ECMO (33). Accordingly, the Extracorporeal Life Support Organization (ELSO) recommends initiating continuous EEG monitoring within 12–24 h of ECMO cannulation and continuing for at least 24–48 h (22). Abnormal EEG patterns may reflect altered cerebral perfusion, ECMO-related hemodynamic changes, or acute brain injury (14, 34, 35). In addition, EEG markers may identify hemispheric asymmetry, focal suppression, and abnormal discharges, which may provide information about neurological risk (7, 33). In comparison to MRI, continuous EEG can be readily and safely initiated soon after cannulation and monitor cerebral activity in real time (22, 33, 36). Multiple single-center and multicenter studies have shown that routine EEG monitoring during ECMO is feasible and safe (14, 16, 37).

Advanced data-driven approaches that integrate EEG dynamics with clinical variables may provide a more objective and sensitive framework for early SNI risk stratification in ECMO patients. This rationale motivated us to explore the fusion model of combining clinical variables and time-dependent EEG spectral features to estimate patient-level SNI risk.

Though our primary hypothesis was refuted, this exploratory study yields three key takeaways. First, incorporating low-temporal-resolution, low-frequency (only delta and theta bands) processed EEG features failed to boost SNI predictive performance, implying future work needs higher-resolution or more informative EEG representations to capture cerebral dynamic changes. Second, only six routine clinical variables delivered decent predictive power for SNI classification, offering a streamlined feature pool for subsequent predictive modeling. Lastly, SHAP-based feature contribution analysis pinpointed high-risk predictors of SNI (Table 2), providing actionable clinical references for risk stratification and diagnosis in pediatric ECMO cohorts.

### 5.2 Evaluation metrics in this study

We selected AUROC as the primary performance metric because it evaluates how well the model discriminates between patients with and without SNI across all classification thresholds. We also emphasized sensitivity because, in this clinical setting, the priority is to identify patients at risk for neurologic injury. For children supported on ECMO, missing a true SNI case may delay neurologic evaluation, monitoring, or intervention, whereas a false-positive prediction would more likely lead to increased clinical attention (38). Specificity, precision, or PPV, and accuracy were therefore considered secondary metrics, as they are useful for describing model performance but less directly aligned with the clinical priority of avoiding missed high-risk cases. Accuracy may also be misleading in imbalanced clinical datasets. Overall, AUROC and sensitivity were prioritized as major evaluation metrics because they best reflect the intended clinical use of the model for the SNI risk-stratification tool.

### 5.3 Comparison of model performance between this study and others

Our MLP model achieved peak average AUROC using only six clinical variables for SNI prediction. To contextualize this performance, we benchmarked against comparable machine learning studies restricted to pediatric ECMO cohorts. This is because pediatric and adult ECMO patients differ drastically in brain maturation, autoregulation, pathophysiology, and brain injury risk, so cross-population comparisons are unreliable. Only two prior works were found to meet our inclusion criteria (pediatric ECMO population + ML for brain injury prediction). Table 3 summarizes head-to-head model comparisons with our work.

**Table 3:** Comparisons of model performance and selected input variables in our study versus other studies for brain injury prediction in pediatric ECMO patients.

Citation	Year	AUROC (%)	Number of patients	Input features
(17)	2020	76.00	174 pediatric ECMO patients	35 temporal clinical and ECMO variables collected from 24 h before ECMO cannulation through the ECMO course. These 35 features include, but are not limited to, systolic blood pressure, diastolic blood pressure, mean blood pressure, heart rate, temperature, platelet count, fibrinogen, hemoglobin, pre-/post-oxygenator pressure, pressure at volume sensor, measured flow, etc.
(19)	2025	91.20 (80.70% on external dataset)	1,633 patients for model development/internal validation; 154 patients for external validation	15 clinical and ECMO variables: Height, age, worst heart rate before ECMO installation, lowest diastolic blood pressure before installation, worst bicarbonate 6 h before installation, worst lactate 6 h before installation, worst partial pressure of carbon dioxide 6 h before installation, extracorporeal cardiopulmonary resuscitation indication, septicemia, cardiac arrest before ECMO, percutaneous catheter method, surgical-incision catheter method, thrombus machine complication, bleeding complications, and other bleeding-related complications.
Our study	/	74.29	43 pediatric ECMO patients	6 clinical variables: age, sex, diagnosis, BSA, weight, and ECMO type.

Reference (17) leveraged 35 temporal clinical and ECMO-related parameters spanning pre-cannulation (24 h prior) to the full ECMO run, while our model attained a comparable AUROC (74.29% vs. 76%) using merely six pre-cannulation clinical covariates. Gathering their 35 multi-source variables carries practical hurdles including frequent missing data. This indicates a streamlined feature set delivers robust predictive power for pediatric ECMO SNI detection. Our readily accessible input variables enhance clinical practicality, cut data collection workload, and facilitate real-world deployment.

Reference (19) reported a higher AUROC than our model but required cumbersome multi-stage preprocessing that elevates complexity and overfitting risk. Their workflow involved removing patients and variables with heavy missing data, deep learning-based data value imputation, and multi-round feature filtering. While this pipeline is methodologically rigorous, its complex preprocessing likely triggered overfitting, evidenced by marked AUROC degradation from internal to external validation. By comparison, our compact, easily acquired clinical feature set delivers a simpler SNI prediction framework for pediatric ECMO care, especially useful in routine clinical settings lacking complete pre-ECMO lab, hemodynamic and complication records.

### 5.4 Feature attribution analysis via SHAP

In the SHAP analysis, diagnosis had the largest absolute SHAP value among all input features, indicating the strongest overall contribution to model-estimated SNI risks. Across the diagnostic categories, cardiac arrest and cardiogenic shock exhibited the strongest positive contributions towards higher estimated SNI risk, suggesting that patients with these clinical indications may be at greater risk of SNI according to the model. In addition, the clinical variables generally exhibited higher SHAP values than the EEG inputs, suggesting that clinical features contributed substantially to the classification of SNI risk in this cohort.

For the EEG inputs, all six relative delta and theta power temporal features had non-zero mean absolute SHAP values (Figure 5), indicating a certain level of contribution toward SNI predictions. However, their overall contributions were relatively lower than those of the clinical variables, suggesting that the selected EEG power temporal features played a less important role in SNI classification within the proposed model.

### 5.5 EEG recording timing and feature selection

EEG monitoring was initiated 6–12 h post ECMO cannulation. Early recording standardizes acute-phase cerebral assessment and enables timely risk stratification free from confounding later interventions or complications. This window also captures EEG markers indicative of acute brain dysfunction in vulnerable ECMO patients (22, 33).

Our multimodal fusion model is engineered for cross-site deployment instead of single-center overfitting, a critical design choice for multicenter EEG datasets. Models overly dependent on absolute EEG power suffer severe domain shifts: hardware, electrode montages, referencing and recording protocols create large inter-site signal discrepancies (39, 40), degrading predictive performance on external cohorts. In contrast, normalized relative EEG power eliminates amplitude biases from electrodes and recording setups, preserving cerebral dysfunction biomarkers while boosting cross-site stability. Our fusion pipeline using these normalized features resists domain shifts and is better adapted for multicenter EEG data.

We analyzed only delta and theta relative power, as low-frequency EEG signals strongly correlate with cerebral dysfunction and neurological damage (41–44). Elevated delta-theta activity is typical in hypoxic-ischemic and diffuse brain injuries (45, 46), and prior work links these bands to injury severity (41, 47–50), justifying our band selection. Alpha, beta and gamma bands were excluded. Their physiological meaning is ambiguous in encephalopathic pediatric ECMO patients, and high-frequency signals are prone to electromyographic contamination, motion and device artifacts (51, 52). Noisy, weakly relevant features degrade model performance in small cohorts; eliminating redundant inputs boosts accuracy, lowers complexity and improves interpretability (53). Given our limited sample size, we retained only robust, physiologically meaningful low-frequency features.

### 5.6 EEG segmentation length versus AUROC

We used a 30-minute segmentation window for our fusion model. We benchmarked alternative window lengths via 7-fold cross-validation across three random seeds, with AUROC outcomes summarized in Supplementary Figure S1. The 30-minute window delivered the best predictive performance. Longer windows reduce valid training segments, while 15-minute windows contain less physiological signal and more noise. The 30-minute duration optimally balances data quantity and signal quality.

However, our 30-minute uninterrupted non-overlapping EEG windows likely induced selection bias, excluding unstable patients with (1) fragmented recordings, (2) severe early neurological injury, or (3) poor electrode contact. We contrasted clinical profiles of the 43 included and 18 excluded patients (Supplementary Table S5, Section A of Supplementary Material). Included patients had significantly older age, larger body surface area, and weight; other metrics showed no statistical differences, yet clinically relevant bias cannot be ruled out. Specifically, 66% of excluded subjects were neonates under 30 days (12/18), versus only 35% (15/43) of included patients. Neonatal EEG recording carries inherent technical hurdles: small scalp area complicates electrode placement, while bedside care and handling worsen artifacts and signal loss (54, 55). ECMO machinery further introduces electrical interference that impairs EEG quality.

While selection bias likely affects younger, smaller patients, gap-free segments were still required in the current to avoid spurious artifact-driven model learning and preserve reliable EEG signals. Low-quality recordings degrade SHAP feature attribution alongside model robustness, generalization and interpretability. Future work will enhance EEG acquisition and expand balanced age-group cohorts for better cross-population performance.

### 5.7 Limitations of the study and future directions

This study has several key limitations. First, it is a single-center study with a small filtered sample size (43 of 73 patients). Discontinuous EEG recordings caused by artifacts and clinical breaks, plus our requirement for 30-min gap-free segments, introduced selection bias biased toward larger children. We plan to enroll 300 pediatric ECMO patients to validate our fusion model, alongside semi/self-supervised learning and improved EEG acquisition to reduce bias. Second, sedatives and antiseizure drugs may distort EEG spectra and confound feature–injury associations. Future work should adjust for medication timing, dosage, sedation depth, anemia, PaCO₂ and oxygenation. Though rare electrographic seizures barely affected model performance, they should be integrated into subsequent training. Third, informative EEG metrics (high-frequency bands, suppression burden, entropy, Hurst exponent, Shannon energy) were excluded here and merit further exploration. Finally, the model only enables early SNI risk stratification, without identifying injury timing, mechanisms or long-term neurodevelopmental outcomes. Longitudinal studies are needed to verify its prognostic value for neurodevelopment.

## 6 Conclusion

This exploratory study developed three frameworks for patient-level SNI risk stratification among pediatric ECMO patients with two focused models: a clinical-only MLP and a multimodal fusion model combining clinical covariates with time-series EEG spectral features. The six-feature clinical MLP attained the maximum AUROC (74.3%) of all evaluated models. This concise, readily obtainable feature set simplifies SNI prediction, proving particularly useful in routine clinical settings without comprehensive pre-ECMO laboratory, hemodynamic, and complication data. On the other hand, merging EEG spectral time series with clinical variables did not enhance predictive performance, plausibly resulting from suboptimal EEG segment screening and selection, low-temporal or low-frequency resolution of EEG inputs, and limited sample volume. Subsequent research needs to enroll larger cohorts, incorporate high-temporal-resolution EEG recordings, explore alternative EEG feature encodings, and conduct multi-site validation to test model generalizability. Upon rigorous external validation, EEG-enhanced risk stratification tools may enable prompt clinical intervention and advance neuroprotective management for pediatric ECMO populations.

## Data Availability

The raw data supporting the conclusions of this article will be made available by the authors, without undue reservation.
