# Supplementary material for: Exploratory deep-learning-driven early risk stratification of significant neurological injury in pediatric extracorporeal membrane oxygenation
Source: Front Digit Health. 2026 Aug 21;8:1857835. doi: 10.3389/fdgth.2026.1857835 (PMC13541705; doi:10.3389/fdgth.2026.1857835)
Supplement: Supplementary file 1 [file Datasheet1.docx]

***Supplementary Material***

A. **Data Description**

**Table S1.** Patient characteristics in the study

Continuous variables are reported as median [IQR]. Categorical variables are reported as n (%), with percentages calculated using non-missing denominators. P values: Mann–Whitney U test for continuous variables; Fisher's exact test for 2-level categorical variables; chi-square test for categorical variables with >2 levels. Abbreviations: BSA, body surface area; ECMO, extracorporeal membrane oxygenation; EEG, electroencephalogram; LOS, length of stay; VA, venoarterial; VV, venovenous.

| Characteristic | Overall (N=43) | No SNI (n=30) | SNI (n=13) | P-value |
| --- | --- | --- | --- | --- |
| Demographics | | | | |
| Age (years) | 1.8 [0, 13.3] | 0.9 [0.0, 11.9] | 4.5 [1.2, 13.5] | 0.22 |
| Female sex, n (%) | 25 (58.1%) | 17 (56.7%) | 8 (61.5%) | 1.00 |
| Weight (kg) | 12.0 [4.0, 56.3] | 9.6 [3.8, 54.3] | 13.9 [8.0, 56.1] | 0.35 |
| Height (cm) | 88.0 [52.8, 154] | 76.0 [52.5, 150] | 99.0 [77.0, 157] | 0.38 |
| BSA (m²) | 0.54 [0.24, 1.56] | 0.45 [0.24, 1.51] | 0.62 [0.43, 1.56] | 0.42 |
| Primary diagnosis | | | | 0.01 |
| Cardiac Arrest | 7 (16.3%) | 1 (3.3%) | 6 (46.2%) |  |
| Cardiogenic Shock | 3 (7.0%) | 2 (6.7%) | 1 (7.7%) |  |
| Pulmonary Hypertension | 13 (30.2%) | 11 (36.7%) | 2 (15.4%) |  |
| Respiratory Failure | 8 (18.6%) | 7 (23.3%) | 1 (7.7%) |  |
| Sepsis | 12 (27.9%) | 9 (30.0%) | 3 (23.1%) |  |
| ECMO type | | | | 0.13 |
| VA | 32 (74.4%) | 20 (66.7%) | 12 (92.3%) |  |
| VV | 11 (25.6%) | 10 (33.3%) | 1 (7.7%) |  |
| Clinical Course | | | | |
| ECMO duration (hour) | 149 [89.6, 254] | 172 [89.7, 325] | 104 [90.5, 184] | 0.157 |
| Hospital LOS (day) | 41.0 [16.5, 52.5] | 41.0 [21.0, 55.5] | 19.0 [7.0, 44] | 0.03 |
| ICU LOS (day) | 13.0 [9.0, 25] | 15.0 [11.0, 23.8] | 10.0 [7.0, 26] | 0.412 |
| Time from hospital admission to ECMO cannulation (hour) | 11.8 [21.2, 44] | 6.1 [2.1, 43.8] | 17.5 [8.1,25.8] | 0.644 |
| EEG and Neuroimaging Features | | | | |
| Time from ECMO cannulation to EEG initiation (hour) | 5.9 [4.1,11.6] | 5.8 [4.2, 9.8] | 6.3 [5.3-16.2] | 0.296 |
| EEG duration (hour) | 47.9 [34.7, 85.4] | 49.4 [35.8, 81] | 46.7 [34.2, 86.1] | 0.761 |
| Time from ECMO cannulation to first neuroimaging (day) | 8.3 [6.2, 20.9] | 9.9 [6.6, 25] | 6.5 [1.9-13.8] | 0.280 |

**Table S2.** EEG recorded power bands and clinical variables used in this study.

| Feature | Name | Data Type | Description  (Frontal polar data excluded) |
| --- | --- | --- | --- |
| EEG | Delta relative power (both),  1-4/1-20 Hz, All 1020 (No Fp) | Continuous | Relative delta power  (both hemisphere) |
|  | Delta relative power (left),  1-4/1-20 Hz, Left Hemi (No Fp) | Continuous | Left relative delta power  (left hemisphere) |
|  | Delta relative power (right),  1-4/1-20 Hz, Right Hemi (No Fp) | Continuous | Right relative delta power  (right hemisphere) |
|  | Theta relative power (both),  4-8/1-20 Hz, All 1020 (No FP) | Continuous | Relative theta power  (both hemisphere) |
|  | Theta relative power (left),  4-8/1-20 Hz, Left Hemi (No Fp) | Continuous | Left relative theta power  (left hemisphere) |
|  | Theta relative power (right),  4-8/1-20 Hz, Right Hemi (No Fp) | Continuous | Right relative theta power  (right hemisphere) |
| Clinical | Age in days | Discrete | Patients' age in days |
|  | ECMO type | Discrete | VA or VV |
|  | Sex | Discrete | 1 for Male and 2 for Female |
|  | Diagnosis | Discrete | Patient diagnosis. |
|  | BSA | Continuous | Body surface area (BSA) computed as: $\text{BSA} \left( \text{m}^{\text{2}} \right)=\sqrt{\frac{\text{height} \left( \text{cm} \right)\cdot\text{weight} \left( \text{kg} \right)}{3600}}$ |
|  | weight | Continuous | Patients’ weight in kilograms (Kg) |

**Table S3**. The number of patients with SNI or No-SNI, and the number of segments

with 1800-sec (or 30-min) being the temporal length of the segment.

| Category | Number | SNI (NIS ≥ 8) | No-SNI (NIS < 8) |
| --- | --- | --- | --- |
| Patients | 43 | 13 | 30 |
| Segmentation | 1026 | 364 | 662 |

**Table S4.** The number of SNI and No-SNI patients in the 7-fold cross-validation.

|  | Training Set | | Validation Set | | Testing Set | |
| --- | --- | --- | --- | --- | --- | --- |
| Fold | SNI | No-SNI | SNI | No-SNI | SNI | No-SNI |
| 1-5 | 10 | 23 | 1 | 3 | 2 | 4 |
| 6 | 10 | 22 | 1 | 3 | 2 | 5 |
| 7 | 11 | 22 | 1 | 3 | 1 | 5 |

**Table S5**. The comparison between the included (43 patients) and the segment failed patients (18 patients) after segmentation. p-values < 0.05 were highlighted in bold. For BSA and Weight, the data representation is: median [Q1, Q3].

| Variable | Type | Included (n=43) | Segment failed (n=18) | P-value | Statistical test |
| --- | --- | --- | --- | --- | --- |
| SNI-positive status | binary | 13/43 (30.2%) | 5/18 (27.8%) | 1.000 | Fisher exact |
| BSA | continuous | 0.54 [0.24, 1.56] | 0.22 [0.19, 0.42] | 0.001 | Mann-Whitney U |
| Weight, scaled as used in model | continuous | 0.12 [0.04, 0.56] | 0.03 [0.03, 0.10] | 0.001 | Mann-Whitney U |
| Age group | categorical | 0-30 days: 15/43 (34.9%); 1-5 years: 6/43 (14.0%); 31-365 days: 3/43 (7.0%); >5 years: 19/43 (44.2%) | 0-30 days: 12/18 (66.7%); 1-5 years: 3/18 (16.7%); 31-365 days: 2/18 (11.1%); >5 years: 1/18 (5.6%) | 0.030 | Chi-square |
| Sex | categorical | Female: 25/43 (58.1%); Male: 18/43 (41.9%) | Female: 8/18 (44.4%); Male: 10/18 (55.6%) | 0.403 | Fisher exact |
| ECMO type | categorical | VA: 32/43 (74.4%); VV: 11/43 (25.6%) | VA: 11/18 (61.1%); VV: 7/18 (38.9%) | 0.361 | Fisher exact |
| Diagnosis | categorical | Cardiac Arrest: 7/43 (16.3%); Cardiogenic Shock: 3/43 (7.0%); Pulmonary Hypertension: 13/43 (30.2%); Respiratory Failure: 8/43 (18.6%); Sepsis: 12/43 (27.9%) | Cardiac Arrest: 1/18 (5.6%); Cardiogenic Shock: 2/18 (11.1%); Pulmonary Hypertension: 9/18 (50.0%); Respiratory Failure: 5/18 (27.8%); Sepsis: 1/18 (5.6%) | 0.183 | Chi-square |

B. **Hyperparameter settings**

**Table S6.** Proposed DenseNet structure: h and w represent the height and width of the input matrix, respectively**.** B: batch size. gr: growth rate.

| Module | Block | Layer | Output dimension | Details |
| --- | --- | --- | --- | --- |
| EEG Feature Extractor | EEG Input | - | (B, h, w, 1) | EEG image-like input |
|  | Initial Convolitional Layer | Conv2D | (B, h, w, 32) | Kernel=(3,3), padding=same, use_bias=False |
|  |  | ReLU | (B, h, w, 32) | - |
|  | Dense Block 1 (L=2) | Dense layer ×2 | (B, h, w, 64) | Each dense layer: Conv(1×1, 4·gr) → Conv(3×3, gr) → Concatenate with input |
|  | Transition 1 | Conv2D | (B, h, w, 32) | Kernel=(1,1), padding=same. |
|  |  | AvgPool2D | (B, h, w, 32) | pool_size=(2,2), stride=1, padding=same |
|  | Dense Block 2 (L=2) | Dense layer ×2 | (B, h, w, 64) | Each dense layer: Conv(1×1, 4·gr) → Conv(3×3, gr) → Concatenate with input |
|  | Transition 2 | Conv2D | (B, h, w, 32) | Kernel=(1,1), padding=same |
|  |  | AvgPool2D | (B, h, w, 32) | pool_size=(2,2), stride=1, padding=same |
|  | Dense Block 3 (L=2) | Dense layer ×2 | (B, h, w, 64) | Each dense layer: Conv(1×1, 4·gr) → Conv(3×3, gr) → Concatenate with input |
|  | Transition 3 | Conv2D | (B, h, w, 32) | Kernel=(1,1), padding=same |
|  |  | AvgPool2D | (B, h, w, 32) | pool_size=(2,2), stride=1, padding=same |
|  | Dense Block 4 (L=2) | Dense layer ×2 | (B, h, w, 64) | Each dense layer: Conv(1×1, 4·gr) → Conv(3×3, gr) → Concatenate with input |
|  | GlobalAvgPool2D | GlobalAvgPool2D | (B, 64) | Global average pooling is used to produce EEG embedding |
| Clinical Feature Extractor | Clinical/tabular input | - | (B, 6) | Clinical/tabular input |
|  | FC1 | Dense | (B, 8) | ReLU |
|  | FC2 | Dense | (B, 16) | ReLU |
|  | FC3 | Dense | (B, 8) | ReLU |
| Binary Classification Head | Fusion | Concatenate | (B, 72) | EEG embedding concatenated with clinical embedding |
|  | Dropout | Dropout | (B, 72) | Rate = 0.2 |
|  | FC1 | Dense | (B, 8) | ReLU |
|  | Output | Dense | (B, 1) | Sigmoid |

**Table S7.** Hyperparameter settings for models that are used in clinical-only input settings. LR: learning rate. ILR: initial learning rate. n_estimator: number of estimators. n_neighbors: number of neighbors.

| Models | Parameter 1 | Selected | Parameter 2 | Selected |
| --- | --- | --- | --- | --- |
| XGBoost | LR: [default, ${10}^{-3}$, ${10}^{-4}$, ${10}^{-5}$] | 10^-4^ | n_estimator:  [200, 400, 800, 1600, 3200] | 1600 |
| GB | LR: [default, ${10}^{-3}$, ${10}^{-4}$, ${10}^{-5}$] | default | n_estimator:  [200, 400, 800, 1600, 3200] | 200 |
| LGBM | LR: [default, ${10}^{-3}$, ${10}^{-4}$, ${10}^{-5}$] | default | n_estimator:  [200, 400, 800, 1600, 3200] | 200 |
| KNN | n_neighbors: [5, 10, 15, 20] | 5 | - | - |
| ANN | ILR: [${10}^{-3}$, ${10}^{-4}$, ${10}^{-5}$] | 10^-3^ | batch size: [4, 32, 64, 128] | 4 |

**Table S8.** Learning rate and batch size settings for models that are used in EEG-only input settings. ILR: initial learning rate.

|  | Parameter 1 | Selected | Parameter 2 | Selected |
| --- | --- | --- | --- | --- |
| Proposed (DenseNet) | ILR: [${10}^{-3}$, ${10}^{-4}$, ${10}^{-5}$] | 10^-3^ | batch size:  [4, 32, 64, 128] | 4 |
| EEGNet | ILR: [${10}^{-3}$, ${10}^{-4}$, ${10}^{-5}$] | 10^-4^ | batch size:  [4, 32, 64, 128] | 128 |
| ShallowConvNet | ILR: [${10}^{-3}$, ${10}^{-4}$, ${10}^{-5}$] | 10^-4^ | batch size:  [4, 32, 64, 128] | 64 |
| EEG-ITNET | ILR: [${10}^{-3}$, ${10}^{-4}$, ${10}^{-5}$] | 10^-3^ | batch size:  [4, 32, 64, 128] | 64 |

**Table S9.** Learning rate and batch size settings for models that are used in dual input settings. ILR: initial learning rate.

|  | Parameter 1 | Selected | Parameter 2 | Selected |
| --- | --- | --- | --- | --- |
| Proposed (DenseNet) | ILR: [${10}^{-3}$, ${10}^{-4}$, ${10}^{-5}$] | 10^-3^ | batch size: [4, 32, 64, 128] | 4 |
| EEGNet | ILR: [${10}^{-3}$, ${10}^{-4}$, ${10}^{-5}$] | 10^-3^ | batch size: [4, 32, 64, 128] | 4 |
| ShallowConvNet | ILR: [${10}^{-3}$, ${10}^{-4}$, ${10}^{-5}$] | 10^-3^ | batch size: [4, 32, 64, 128] | 4 |
| EEG-ITNET | ILR: [${10}^{-3}$, ${10}^{-4}$, ${10}^{-5}$] | 10^-3^ | batch size: [4, 32, 64, 128] | 4 |

**Table S10.** Hyperparameter settings for EEGNet.

| Hyperparameter | Setting |
| --- | --- |
| F1 | 8 |
| D | 2 |
| F2 | 16 |
| Kerne length | 64 |
| Dropout Rate | 0.25 |
| Norm Rate | 0.25 |
| Pooling size 1 | 4 |
| Pooling size 2 | 8 |
| Separable kernel length | 16 |

**Table S11.** Hyperparameter settings for EEG-ITNet.

| Hyperparameter | Setting |
| --- | --- |
| Inception filters | (2, 4, 8) |
| Inception kernel sizes | (16, 32, 64) |
| Inception depth multiplier | 1 |
| Pool size after inception block | 4 |
| Temporal convolution kernel size | 4 |
| Temporal convolution dilations | (1, 2, 4, 8) |
| Dropout rate | 0.2 |
| Dimension-reduction filters | 14 |
| Pool size after DR block | 4 |

**Table S12.** Hyperparameter settings for ShallowConvNet.

| Hyperparameter | Setting |
| --- | --- |
| Number of temporal filters | 40 |
| Temporal filter length | 25 |
| Mean pooling size | 75 |
| Mean pooling stride | 15 |
| Dropout rate | 0.5 |

**Figure S1**. Model loss of the proposed DenseNet framework under different segmentation lengths. The loss was calculated using binary cross-entropy, with lower values indicating better model performance and higher values indicating worse performance.
